# High molecular weight of polysaccharides from *Hericium erinaceus* against amyloid beta-induced neurotoxicity

**DOI:** 10.1186/s12906-016-1154-5

**Published:** 2016-06-07

**Authors:** Jai-Hong Cheng, Chia-Ling Tsai, Yi-Yang Lien, Meng-Shiou Lee, Shyang-Chwen Sheu

**Affiliations:** Department of Medical Research, Center for Shockwave Medicine and Tissue Engineering, Kaohsiung Chang Gung Memorial Hospital and Chang Gung University College of Medicine, Kaohsiung, Taiwan; Department of Food Science, National Pingtung University of Science and Technology, No. 1, Shuehfu Rd, Neipu, Pingtung Taiwan; Department of Veterinary Medicine, National Pingtung University of Science and Technology, No. 1, Shuehfu Rd, Neipu, Pingtung Taiwan; Department of Chinese Pharmaceutical Sciences and Chinese Medicine Resources, China Medical University, Taichung, Taiwan

**Keywords:** *Hericium erinaceus*, Polysaccharides, Amyloid beta, Neuroprotection, PC12 cell

## Abstract

**Background:**

*Hericium erinaceus* (HE) is a well-known mushroom in traditional Chinese food and medicine. HE extracts from the fruiting body and mycelia not only exhibit immunomodulatory, antimutagenic and antitumor activity but also have neuroprotective properties. Here, we purified HE polysaccharides (HEPS), composed of two high molecular weight polysaccharides (1.7 × 10^5^ Da and 1.1 × 10^5^ Da), and evaluated their protective effects on amyloid beta (Aβ)-induced neurotoxicity in rat pheochromocytoma PC12 cells.

**Methods:**

HEPS were prepared and purified using a 95 % ethanol extraction method. The components of HEPS were analyzed and the molecular weights of the polysaccharides were determined using high-pressure liquid chromatography (HPLC). The neuroprotective effects of the polysaccharides were evaluated through a 2,2-diphenyl-1-picrylhydrazyl (DPPH) radical scavenging assay and an MTT assay and by quantifying reactive oxygen species (ROS) and mitochondrial membrane potentials (MMP) of Aβ-induced neurotoxicity in cells.

**Result:**

Our results showed that 250 μg/ml HEPS was harmless and promoted cell viability with 1.2 μM Aβ treatment. We observed that the free radical scavenging rate exceeded 90 % when the concentration of HEPS was higher than 1 mg/mL in cells. The HEPS decreased the production of ROS from 80 to 58 % in a dose-dependent manner. Cell pretreatment with 250 μg/mL HEPS significantly reduced Aβ-induced high MMPs from 74 to 51 % and 94 to 62 % at 24 and 48 h, respectively. Finally, 250 μg/mL of HEPS prevented Aβ-induced cell shrinkage and nuclear degradation of PC12 cells.

**Conclusion:**

Our results demonstrate that HEPS exhibit antioxidant and neuroprotective effects on Aβ-induced neurotoxicity in neurons.

## Background

*Hericium erinaceus* (HE) is a well-known mushroom that is consumed as food and used in traditional Chinese medicine. These mushrooms contain physiologically significant components, such as β-glucan polysaccharides and other biomaterials, which have demonstrated anticancer, immunomodulatory, hypolipidemic, antioxidant and neuroprotective properties [1–6]. As an anticancer agent, the polysaccharides from HE have more significant anti-artificial pulmonary metastatic tumor effects and immunomodulatory activity than those of *Hericium laciniatum* [1]. HE and *Lentinus edodes* have been compared with regard to their antitumor activities and immunoregulatory effects on mice with sarcoma 180 [7]. Additionally, HE extracts (HTJ5 and HTJ5A) have been found to be more effective and less toxic than clinically used anticancer drugs such as 5-fluorouracil against liver cancer HepG2 and Huh-7, colon cancer HT-29 and gastric cancer NCI-87 cells in vitro and in tumor xenografts in vivo [8].

Macrophages are activated by HE polysaccharides to produce nitric oxide and express cytokines (IL-1beta and TNF-beta), which lead to effective antitumor activity and immunomodulation [9]. Previously, we demonstrated that HE extracts can induce the activation of dendritic cells and increase the secretion of IL-12 to modulate a TH1 immune response [2]. The hypolipidemic effects proportionally increased with oral administration of an HE exo-biopolymer in a dose-dependent manner in animal studies [3]. The HE biomaterials reduced levels of low-density lipoprotein cholesterol while maintaining relatively high levels of high-density lipoprotein cholesterol and reduced the risk of atherosclerosis.

It was previously reported that HE extracts have neuroprotective effects, promote normal development of cultivated cerebellar cells and have regulatory effects on the development of myelin genesis processes in vitro [10]. The ethanol extract of HE has been shown to induce nerve growth factor expression and to prevent Aβ_25–35_-induced impairment of memory functions in animal experiments [11, 12]. Oxidative stress has been shown to be involved in the initiation and progression of various disorders caused by oxygen radicals, which damages lipids, proteins and nucleic acids [13, 14]. The hot water extract of HE has been reported to improve this free radical scavenging activity and inhibit lipid peroxidation [15]. HE polysaccharide extracts have been reported to decrease lipid peroxidation levels, increase antioxidant enzyme activity and increase radical scavenging activity [4, 16, 17].

In this study, we purified HEPS, which consists of two high molecular weight polysaccharides and exhibits antioxidant activity, from fruiting bodies. HEPS-treated cells showed an increase in the rate of free radical scavenging, a reduction in the production of ROS, a recovery in mitochondrial function, maintenance in morphology changes, and a reduction in cell apoptosis of PC12 cells upon Aβ treatment. Finally, we demonstrated that HEPS has neuroprotective properties for neurons.

## Methods

### Cell culture

PC12 cells were purchased from the Bioresource Collection and Research Center of the Food Industry and Development Research Institute in Taiwan. Cells were grown in RPMI 1640 with 10 % heat inactivated horse serum, 5 % fetal bovine serum, penicillin (50 units/ml), and streptomycin (50 mg/ml). The cells were cultivated in an incubator with 5 % CO_2_ at 37 °C.

### Preparation of HEPS

Fresh fruiting bodies of HE were obtained from a local farm as previously reported [2]. Samples of HE were identified by Professor Wen-Te Chang of China Medical University (CMU) in Taiwan. The HE voucher specimen and number (CPSCMU HE 1021202) were deposited to the School of Chinese Medicine Resources (SCMR) at CMU. A modified procedure from Dr. Mori’s report was used to prepare HEPS [11]. The whole fruiting body was cleaned, lyophilized and powdered. The HE powder was mixed with two volumes of ethanol (95 %) and homogenized at 200 rpm for 1 h. This procedure was repeated three times. The mixture was then filtered with Whatman filter paper (Sigma-Aldrich, USA), and the extract was collected by centrifuging the mixture at 10,000 × g for 10 min at 4 °C. The HEPS supernatant was then lyophilized and stored at −20 °C until used for experiments.

### Measuring components and molecular weights of HEPS

The total sugars and reducing sugars in the extract were measured as previously described [2]. The Bradford method was used to determine the total concentration of protein using a protein assay kit (Bio-Rad, USA) following the manufacturer’s instructions. Flavonoids from HEPS were measured using previously described methods [18]. Flavonoid content curves were determined using quercetin as a standard. The endotoxicity of HEPS was measured using a chromogenic *Limulus amebocyte* lysate kit (Associates of Cape Cod, USA), where the maximum sensitivity level was 0.25 EU/mL [2].

The molecular weights of HEPS components were determined by HPLC analysis. The extract was dissolved in deionized water, filtered through a 0.45 μm membrane and applied to a Hitachi L-2490 HPLC system (Tokyo, Japan) as a 20 μL aliquot. The system was fit with a TSK-GEL G3000PWXL column (7.8 mm × 30 cm) and was maintained at a temperature of 25 °C. The extract was eluted with deionized water at a flow rate of 0.6 mL/min and detected by a refractive index detector (RID). Pullulan standards of various molecular weights (5900, 11,800, 22,800, 47,300, and 112,000 daltons) were used to establish standard curves and to determine molecular weights [2].

### DPPH radical scavenging assay

The free radical scavenging rate was evaluated by measuring the 2,2-diphenyl-1-picrylhydrazyl (DPPH) scavenging activity of HEPS. The DPPH assay used a modified procedure from a previously described study [19]. The HE extracts were dissolved in methanol and mixed with 250 μL of a 0.2 mM DPPH radical solution (Sigma-Aldrich, USA). After 30 min at room temperature, the absorbance of the resulting solutions and a blank were recorded against 0.1 mg/mL butylated hydroxyanisole (BHA) and L-ascorbic acid (Vitamin C; Sigma-Aldrich, USA) as positive controls. The absorbance of each reaction was recorded in triplicate. The disappearance of DPPH radicals was measured spectrophotometrically at 517 nm using a Hitachi U-2001 spectrophotometer (Tokyo, Japan), and the DPPH scavenging effect was calculated as previously described [19].

### MTT assay for cell cytotoxicity and protection

The MTT assay was used for three experiments. First, the cell cytotoxicity of HEPS was measured by plating exponentially growing PC12 cells at a density of 5 × 10^4^ cells/well in 96-well plates, which were exposed with or without 25, 50, 100, 200, 250 μg/mL of HEPS for 24 and 48 h. The second stage of the assay measured cell cytotoxicity of Aβ_1__-__40_ (Sigma-Aldrich, USA) by adding 1.2 μM Aβ_1__-__40_ to PC12 cells for 24 and 48 h. The third stage was a cell protection assay, in which PC12 cells were incubated with 25, 50, 100, 200, 250 μg/mL of HEPS for 24 h, and 1.2 μM Aβ_1__-__40_ was added for 24 and 48 h. After each of these three experiments, the cells were incubated with 2 mg/mL 3-(4,5-dimethylthiazol-2-yl)-2,5-diphenyl tetrazolium bromide (MTT) for 4 h at 37 °C, the media was carefully removed and 100 μl of DMSO was added to each well. Dark blue formazan crystals formed, the intact cells were solubilized for 30 min, and the absorbance at 570 nm was measured with a PowerWave XS ELISA reader (Bio-Tek, USA). The results were expressed as the percentage of MTT reduction, assuming the absorbance of control cells was 100 %.

### ROS and MMP measurements

To measure ROS, cells treated with HEPS and Aβ_1__-__40_ were collected and centrifuged at 650 × g for 10 min. The resulting pellets were washed once with phosphate buffered saline (PBS). These steps were repeated twice. The ROS production rate was measured using an OxiSelect™ Intracellular ROS Assay Kit, and the intracellular accumulation of ROS was monitored using the cell-permeable fluorogenic probe 2’,7’-dichlorodihydrofluorescein diacetate (DCFH-DA).

The MMP was measured using the fluorescent dye JC-1 [20]. Mitochondria with high MMP promoted the formation of J-aggregates and fluoresced red. In contrast, mitochondria with low MMP contained JC-1 monomers and fluoresced green. After co-treating cells with 1.2 μM Aβ_1__-__40_ for 24 h in the presence or absence of HEPS, 1 × 10^6^ cells/mL were collected and incubated for 15 min at 37 °C. JC-1 (10 μg/mL) was then loaded, and the fluorescence intensity of the cells was examined at an excitation of 485 nm and emission of 535 nm using FACScan flow cytometry (Becton Dickinson, USA).

### Cell morphology and intracellular fluorescence staining

The DNA-binding dye acridine orange (Sigma-Aldrich, USA) was used to observe the morphological characteristics of the treated cells. After PC12 cells were incubated with 1.2 μM Aβ_1__-__40_ or HEPS at 37 °C for 24 and 48 h, the cells were washed with sterilized PBS three times and incubated with acridine orange (10 μg/ml) at 37 °C for 10 min in the dark. The stained cells were observed and photographed using an Olympus COVER-018 fluorescence microscope (Tokyo, Japan).

### Statistical analysis

The data were analyzed using Statistical Analysis System (SAS) software (SAS Institute, USA) as described previously [2]. A one-way analysis of variance (one-way ANOVA) and Duncan’s test were used to determine the statistical significance between groups. Differences were considered statistically significant when *p* ≤ 0.05.

## Results and discussion

### The composition and cell toxicity of HEPS

**Fig. 1:**
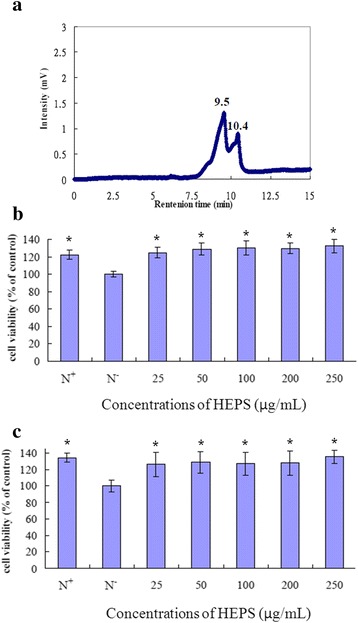
Measuring the molecular weight and toxicity of HEPS. **a** The molecular weight of HEPS was measured by HPLC. **b** 24 and (**c**) 48 h PC12 cell incubation with different concentrations of HEPS (25, 50, 100, 200 and 250 μg/mL, respectively). Cell viability was measured by an MTT assay with HEPS. RPMI-1640 complete medium containing 10 % horse serum and 5 % fetal bovine serum is indicated as N^+^. Serum-free RPMI-1640 medium is indicated as N^−^. All values are mean ± SD and used a one-way analysis of variance (ANOVA, *n* = 6). All columns were significantly different compared with N^−^ and are indicated as * (*p* < 0.05)

The fruiting bodies of HE were cleaned, lyophilized and powdered. The powder of HE was extracted using 95 % alcohol, and lyophilization afforded HEPS. The composition of HEPS was then analyzed as shown in Table 1. The total sugar content was determined to be 311 mg/g, while the reducing sugar content was 249 mg/g. Furthermore, the total amount of protein was 135 μg/g. As previously reported, flavonoids are directly associated with the daily human dietary intake of antioxidants and are important for health benefits, neuroprotection and may potentially delay the development of Alzheimer's disease (AD)-like pathology [21–23]. We determined that the flavonoid concentration in HEPS was 99 ng/g using quercetin as a standard. The molecular weight of HEPS components were measured by HPLC analysis. The retention times were 9.5 and 10.4 min and the molecular weights of the individual components were 1.7 × 10^5^ daltons and 1.1 × 10^5^ daltons, respectively (Fig. 1a). The molecular weights of these major components were different from those in our previous study (2.2 × 10^4^ daltons), which used different purification procedures [2]. Bioactive polysaccharides that are higher in molecular weight have been shown to exhibit significant antitumor properties, immunomodulatory activity, antioxidant activity, and neuroprotection [24, 25]. In this study, we purified the high molecular weight polysaccharides from HE and tested their neuroprotective properties.

**Table 1:** Compositions of the extract from *Hericium erinaceus*

Content	Total sugar	Reducing sugar	Protein	Flavonoids
Extract (mg/g)	311 ± 27.8^a^	249 ± 25.8^a^	135 ± 0.1^a^	99 ± 1.7^a^

^a^The values are shown as mean ± SD (*n* = 3)

**Fig. 2:**
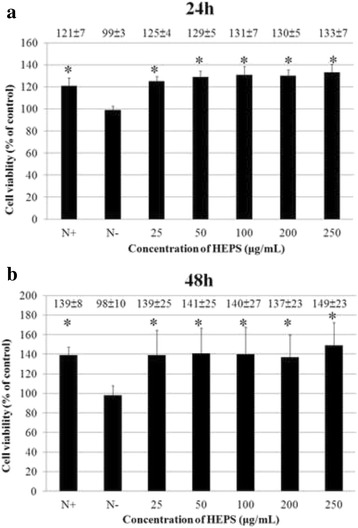
The cell viability of PC12 cells with HEPS. **a**, (**b**) PC12 cells were treated with different concentrations (0, 25, 50, 100, 200 and 250 μg/mL) of HEPS and incubated for 24 and 48 h. Cell viability measurements were used to evaluate cell death using the MTT assay. Serum-free RPMI-1640 medium is indicated as N^−^. All values are mean ± SD and used a one-way analysis of variance (ANOVA, *n* = 6). All columns were significantly different (*p* < 0.05)

Prior to the experiments, endotoxin contamination levels were examined and determined to be lower than 0.25 EU/mL. Results from the cell cytotoxicity assay (MTT assay) are shown in Fig. 1b and c. Different concentrations of HEPS (25 to 250 μg/mL) were added to PC12 cells and incubated for 24 and 48 h. There was no significant difference in cell viability after incubation for 24 h (125, 129, 131, 130 and 133 %) and 48 h (139, 141, 140, 137 and 149 %) with different HEPS concentrations compared to N^+^ (complete medium; 121 ± 7.1 % for 24 h and 139 ± 8.3 % for 48 h) as shown in Fig. 2. The results indicate that HEPS was harmless to PC12 cells.

### HEPS protecting PC12 cells against Aβ_1–40_ induced neurotoxicity

**Fig. 3:**
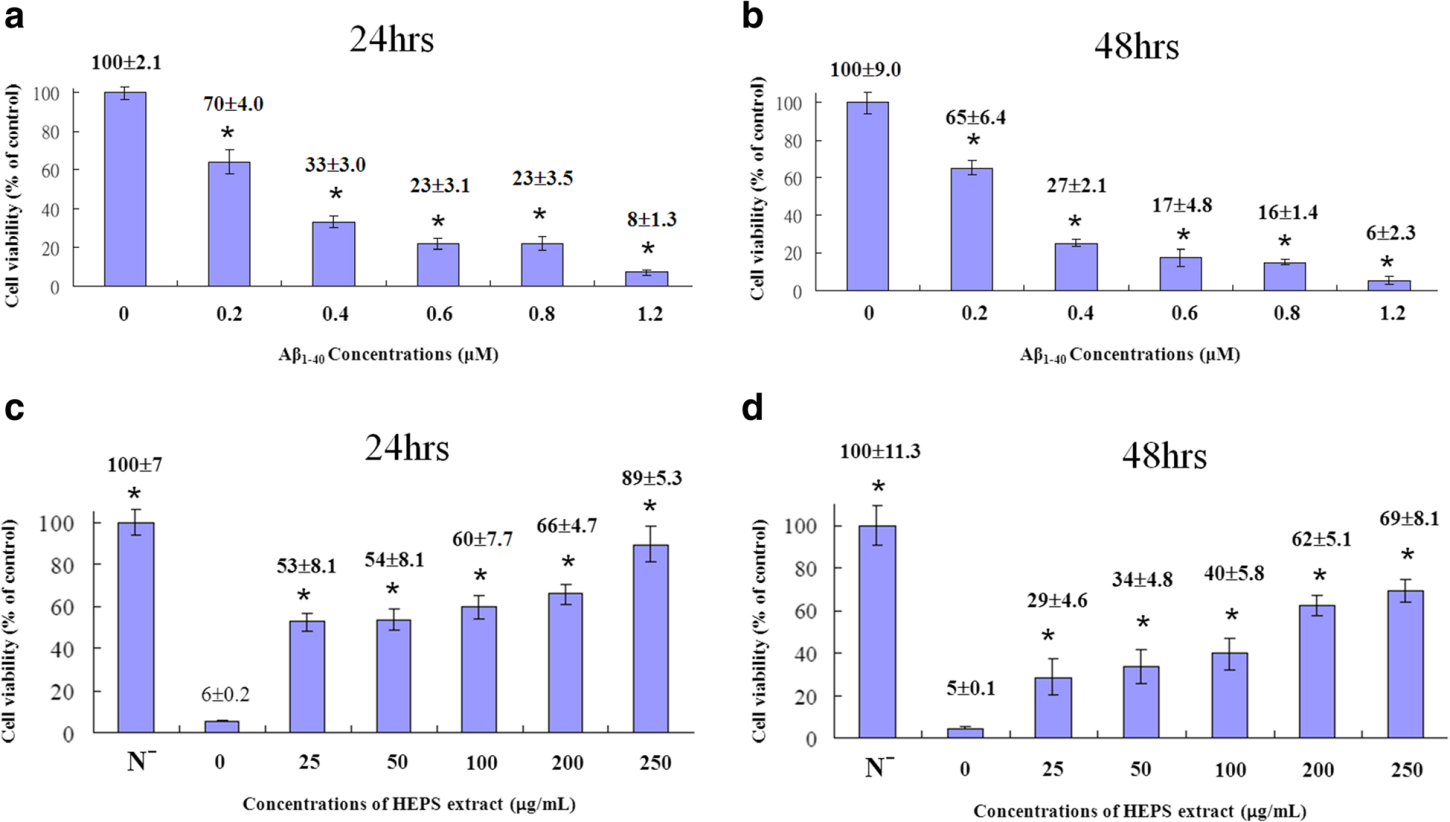
The protective effects of HEPS on Aβ-induced toxicity in PC12 cells. **a**, (**b**) PC12 cells were treated with different concentrations of Aβ_1–40_ (0, 0.2, 0.4, 0.6, 0.8 and 1.2 μM) for 24 and 48 h and the toxicity was analyzed. **c**, (**d**) PC12 cells were treated with different concentrations (0, 25, 50, 100, 200 and 250 μg/mL) of HEPS followed by addition of 1.2 μM Aβ_1–40_ and incubation for 24 and 48 h. Cell viability measurements were used to evaluate cell death using the MTT assay. Serum-free RPMI-1640 medium is indicated as N^−^. All values are mean ± SD and used a one-way analysis of variance (ANOVA, *n* = 6). All columns were significantly different (*p* < 0.05)

Accumulation of Aβ is considered to play a crucial role in the initiation and progression of AD [26, 27]. AD-associated neurotoxic mechanisms include oxidative stress, mitochondrial dysfunction, and apoptosis, which cause abnormal neuronal function. We utilized different concentrations of Aβ_1–40_ to induce cytotoxicity and evaluated cells with the MTT assay. As shown in Fig. 3a and b, 1.2 μM of Aβ_1–40_ significantly decreased cell viability from 100 to 8 % and 6 % after treatment for 24 and 48 h. Nevertheless, we explored the dose-dependent attenuating effects of HEPS pre-treatment on Aβ_1–40_-induced toxicity (Fig. 3c and d). Cell viability improved to 89 and 69 % with 250 μg/mL of HEPS after incubation with Aβ_1–40_ for 24 and 48 h. HEPS concentrations higher than 250 μg/mL were also measured, but there were no improvements in neuroprotective effects (data not shown). Researchers have reported that polysaccharide extracts and their derivatives from parts of plants and mushrooms not only had immunomodulatory abilities but also improved neuronal growth and protection [2, 25, 28–30]. Polysaccharide extracts from *Lycium barbarum* have been shown to have neuroprotective effects against fibrillar Aβ_1–40_ and Aβ_25–35_ fragment toxicity, as well as improved learning, memory and neurogenesis in animal studies [25, 31, 32]. Additional therapeutic effects of HEPS and its derivatives require further studies. Finally, our results demonstrated that HEPS had protective benefits against Aβ cytotoxicity in PC12 cells.

### HEPS inhibited accumulation of free radical and ROS in cells

**Fig. 4:**
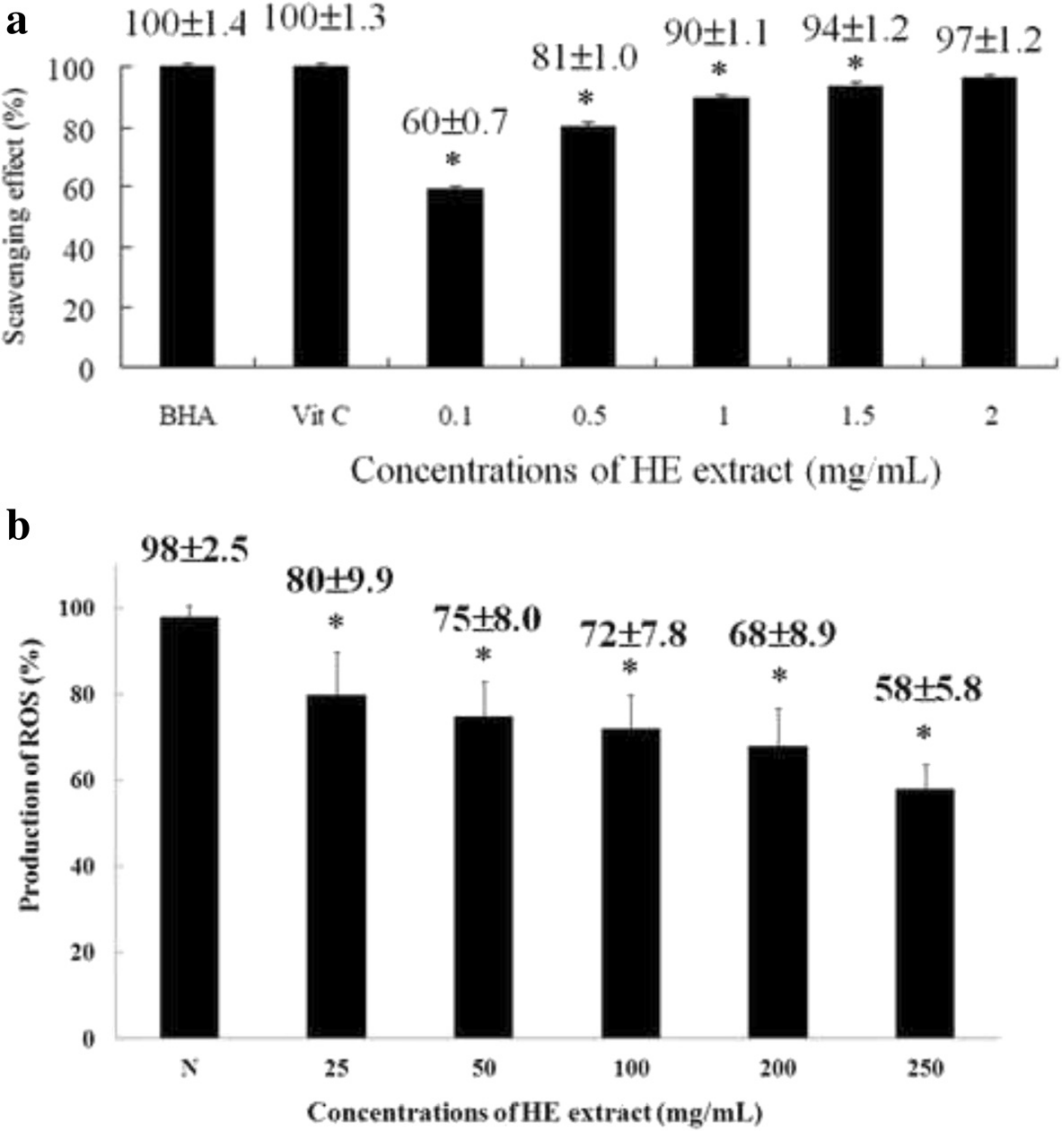
The scavenging activity of HEPS and production of ROS on Aβ-induced neurotoxicity in PC12 cells upon pretreatment of HEPS. **a** Different concentrations (0.1, 0.5, 1, 1.5 and 2 mg/mL) of HEPS were added into PC12 cells the free radical scavenging activity was measured using the DPPH assay. BHA and Vit C (0.1 mg/mL) were used as positive controls. **b** The effect of HEPS (25, 50, 100, 200 and 250 μg/mL) on the production of ROS was followed by 1.2 μM Aβ-induced neurotoxicity in PC12 cells. The production of ROS was measured using an ROS assay kit. N indicates samples without HEPS. All values are mean ± SD and used a one-way analysis of variance (ANOVA, *n* = 3 in panel A and *n* = 6 in panel B). All columns were significantly different (*p* < 0.05)

Polysaccharides extracted from *Antrodia cinnamomea* and other mushrooms have demonstrated antioxidant properties that involve up-regulation of glutathione S-transferase (GST) activity, maintenance of normal glutathione (GSH)/ oxidized glutathione (GSSG) ratios, and scavenging of ROS [33]. In order to survey the antioxidant activity of HEPS, DPPH assays were carried out to measure free radical scavenging. Different concentrations of HEPS (0.1, 0.5, 1, 1.5 and 2 mg/mL) were added into PC12 cells and the scavenging effects were monitored (Fig. 4a). BHA and Vit C were added as positive controls. Over 90 % of scavenging free radicals were detected at 1 mg/mL of HEPS. The highest quantity of scavenging free radicals (97 %) was detected at 2 mg/mL of HEPS. As previously reported, HEPS cultivated in Malaysia exhibited antioxidant properties and over a 90 % scavenging effect at 7 mg/mL [4]. Selenium-containing polysaccharides in HE shake flask cultures reached 100 % antioxidant activity at 5 mg/mL while an absence of selenium exhibited a less pronounced antioxidant activity of 72 % at the same concentration [34]. These results indicate that our methods of preparing HEPS produced polysaccharides with greater antioxidant activity.

Mitochondria are a major source of ROS, which are produced in many normal and abnormal physiological processes [35]. However, excessive ROS production may cause damage during the accumulation of Aβ in the pathogenesis of AD [36]. As shown in Fig. 4b, pretreatment of HEPS at concentrations ranging from 25 μg/mL to 250 μg/mL significantly decreased the production of ROS from 80 to 58 % after Aβ incubation for 24 h. Moreover, 250 μg/mL HEPS considerably reduced ROS levels to 40 % compared to cells without HEPS pretreatment, suggesting that HEPS protects mitochondria and reduces ROS generation.

### HEPS prevents loss of MMP in PC12 cells

**Fig. 5:**
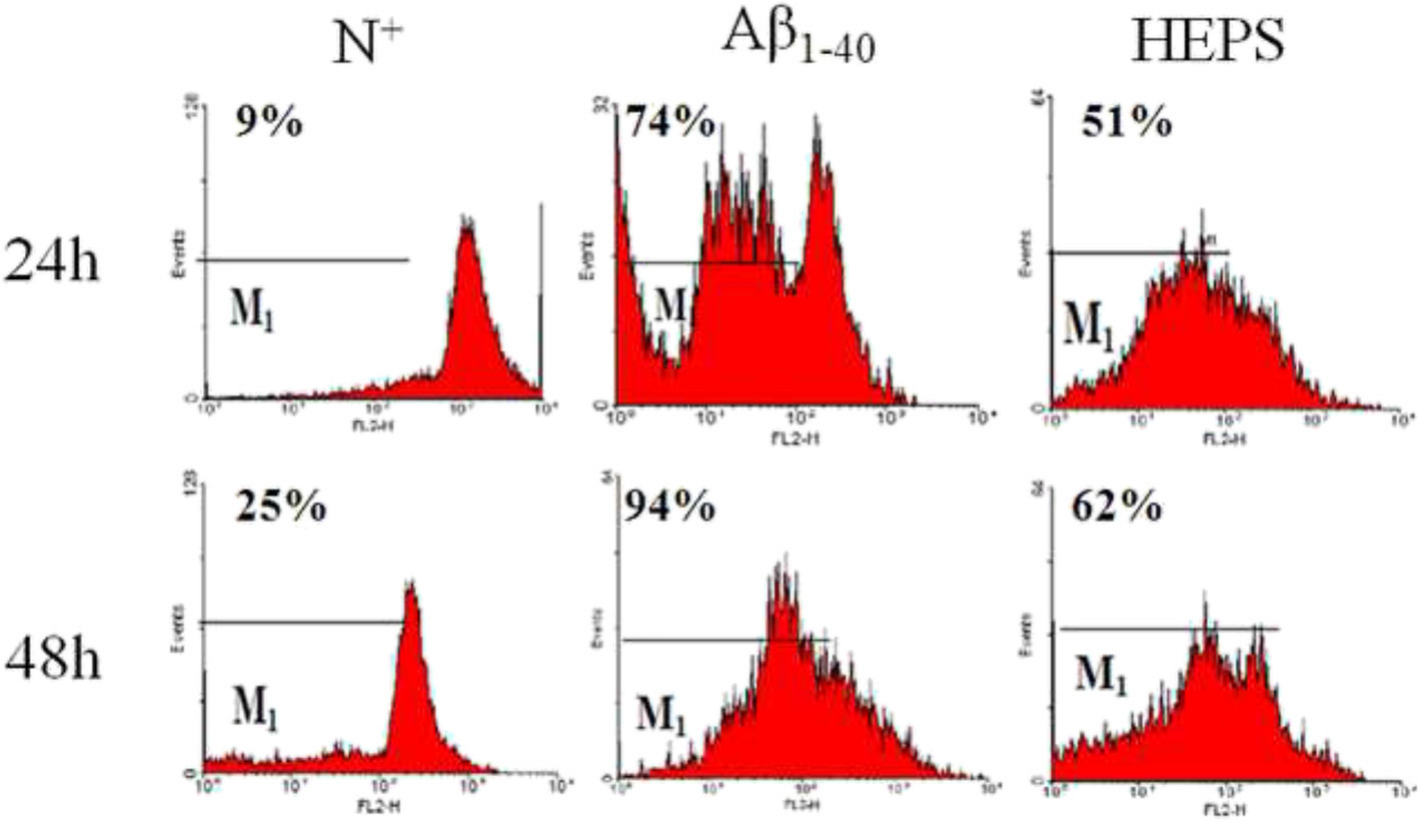
Measurement of MMP in PC12 cells. Cells were incubated with 1.2 μM Aβ_1–40_ for 24 and 48 h and MMP changes were measured using FACScan flow cytometry. MMPs were then reduced by pretreating samples with 250 μg/mL HEPS followed by 1.2 μM Aβ_1–40_. RPMI-1640 complete medium containing 10 % horse serum and 5 % fetal bovine serum is indicated as N^+^. Cells were incubated with 1.2 μM Aβ_1–40_ for 24 and 48 h, and MMP changes were measured using FACScan flow cytometry. MMPs were reduced by pretreating samples with 250 μg/mL HEPS followed by 1.2 μM Aβ_1–40_. RPMI-1640 complete medium containing 10 % horse serum and 5 % fetal bovine serum are indicated as N^+^

Growing evidence suggests that high Aβ levels result in mitochondrial abnormalities through a mechanism that is not clearly established [37]. Both the amyloid precursor protein (APP) and Aβ have been found in mitochondrial membranes and interact with mitochondrial proteins. Overproduction of these proteins has been found to increase interruptions in electron transfer and to impair mitochondrial function [38, 39]. To further examine the protective effect of HEPS in mitochondrial function, we measured the loss of mitochondrial membrane potential using the JC-1 dye to functionally stain mitochondria in PC12 cells [20]. Cells were treated with 1.2 μM Aβ_1–40_ for 24 and 48 h, and flow cytometry indicated that MMPs were reduced and the green fluorescence of JC1 monomer increased to 74 and 94 % in PC12 cells (Fig. 5). Pretreatment with HEPS showed that mitochondrial functions were protected, and MMP recovery decreased to 51 and 61 % after 24 and 48 h of Aβ incubation. Dr. Eckert reported that *Ginkgo biloba* extract EGb 761 significantly improved the MMP of PC12 cells in a dose dependent manner and also treated age-related cognitive disorders such as AD [40]. Our results show that Aβ_1–40_ changed mitochondrial function and that HEPS protected against Aβ_1–40_-induced abnormalities in the MMP of mitochondria in PC12 cells.

### Measurement of morphology and intracellular changes

**Fig. 6:**
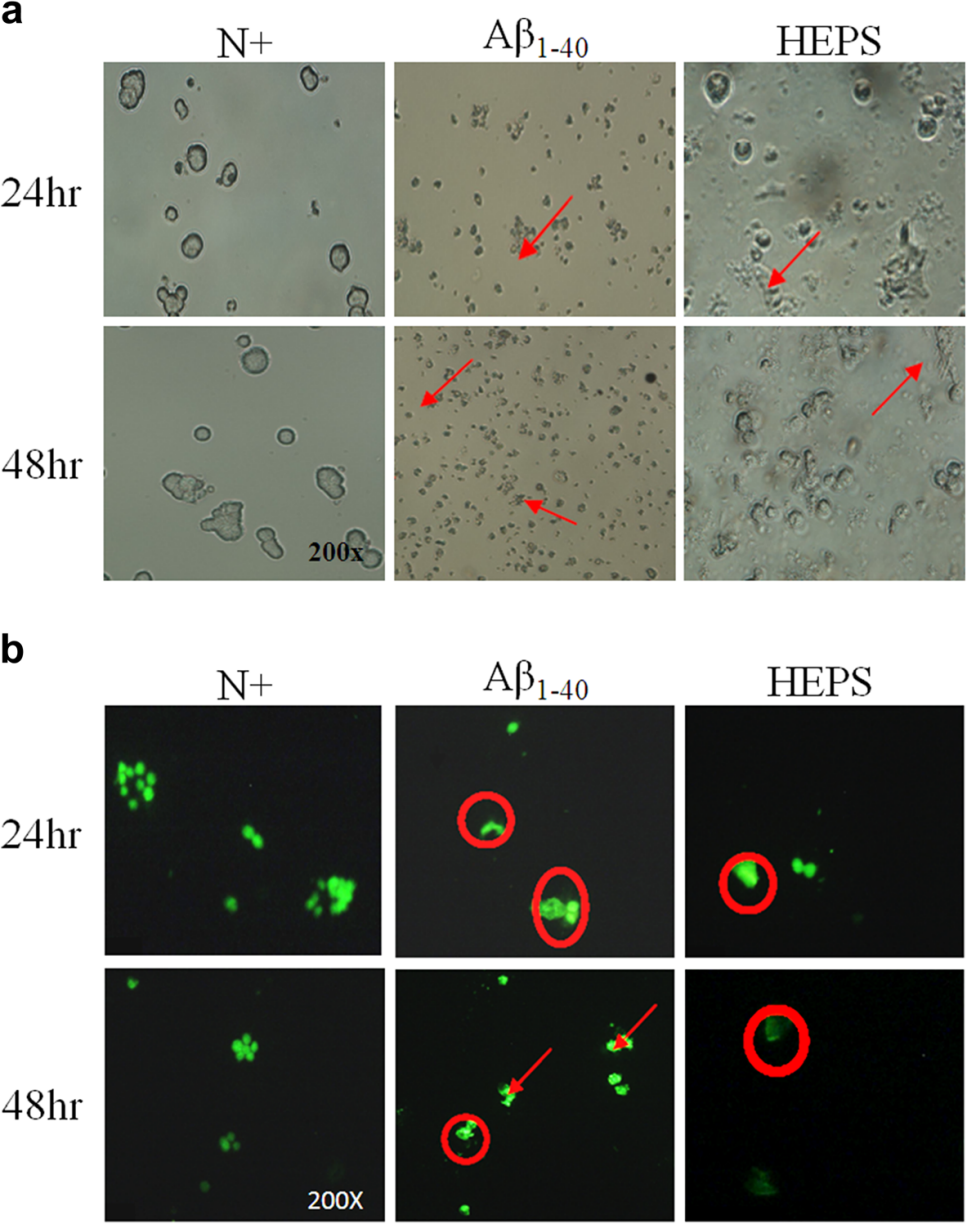
Morphological changes in PC12 cells were induced by Aβ, and cells were protected by pretreatment with HEPS. **a** Morphological changes in PC12 cells were observed by microscope after incubation with 1.2 μM Aβ_1–40_ for 24 and 48 h. **b** PC12 cells were protected by HEPS (250 μg/mL) followed by incubation with 1.2 μM Aβ_1–40_ for 24 and 48 h. Intracellular fluorescence was observed by fluorescence microscopy. RPMI-1640 complete medium containing 10 % horse serum and 5 % fetal bovine serum are indicated as N^+^. Circles and arrows indicate nuclear fragments and cell rupture, respectively

The aggregation of Aβ_1–40_ induces neuronal damage such as the breakdown of oligodendrocytes and the emergence of shrunken cell bodies [41]. PC12 cells ruptured and shrunk with Aβ_1–42_ treatment after 24 and 48 h (Fig. 6a and b; Aβ_1–42_ treatment). However, these damages were less significant in cell cultures containing 250 μg/mL HEPS followed by incubation with Aβ_1–42_ (Fig. 6a and b; HEPS treatment). We also used acridine orange staining to observe fragmentation and rupture of cellular nuclei, which formed apoptotic bodies (Fig. 6b; comparing Aβ_1–42_ and HEPS). Pretreatment with HEPS reduced PC12 cell apoptosis and decreased cellular damage. These observations suggest that HEPS could reduce cell apoptosis and may have the ability to protect cells.

The compound CBNU06 is purified from *Isodon japonicas* and protects PC12 cells from Aβ-induced neurotoxicity and reduces the number of cells that undergo DNA condensation and fragmentation by inhibiting NF-kB signaling pathways [42]. *Atractylodes macrocephala* polysaccharides have demonstrated neuroprotective effects by decreasing the expression of Bax and Caspase-3 and increasing Bcl-2 levels in neurons [43]. However, the actual mechanism of protecting and reducing cell apoptosis by HEPS needs further investigation.

## Conclusions

Our results demonstrate that pretreatment of PC12 cells with HEPS, which contains two high molecular weight polysaccharides, promotes antioxidant activity and has neuroprotective effects against on Aβ-induced neurotoxicity. We show that HEPS promoted cell viability under Aβ-induced toxic conditions. Furthermore, HEPS also increased the efficacy of free radical scavenging and ROS. Finally, HEPS protected PC12 cells against Aβ-induced cell apoptosis. In summary, our previous and current findings suggest that different molecular weight polysaccharides from HE not only play a role in immunomodulation of dendritic cells but also contain neuroprotective effects for neurons.

## Abbreviations

AD, Alzheimer's disease; APP, amyloid precursor protein; Aβ, amyloid beta; BHA, butylated hydroxyanisole; DCFH-DA, 2’, 7’-Dichlorodihydrofluorescin diacetate; DPPH, 2,2-diphenyl-1-picrylhydrazil; GSH, glutathione; GSSG, oxidized glutathione; GST, glutathione S-transferase; HE, Hericium erinaceus; HPLC, high pressure liquid chromatography; MMP, mitochondrial membrane potential; MTT, 3- (4,5-cimethylthiazol-2-yl)-2,5-diphenyl tetrazolium bromide; PBS, phosphate buffered saline; RID, refractive index detector; ROS, reactive oxygen species; SAS, Statistical Analysis System
